# Ultra-sensitive Pressure sensor based on guided straight mechanical cracks

**DOI:** 10.1038/srep40116

**Published:** 2017-01-06

**Authors:** Yong Whan Choi, Daeshik Kang, Peter V. Pikhitsa, Taemin Lee, Sang Moon Kim, Gunhee Lee, Dongha Tahk, Mansoo Choi

**Affiliations:** 1 Global Frontier Center for Multiscale Energy Systems, Department of Mechanical and Aerospace Engineering, Seoul National University, Seoul 151-742, Korea; 2 Department of Mechanical Engineering, Ajou University, San 5, Woncheon-dong, Yeongtong-gu, Suwon 443-749, Republic of Korea; 3 Division of WCU Multiscale Mechanical Design, Department of Mechanical and Aerospace Engineering, Seoul National University, Seoul 151-742, Korea; 4 Department of Mechanical Engineering, Incheon National University, Incheon, 406-772, Korea

## Abstract

Recently, a mechanical crack-based strain sensor with high sensitivity was proposed by producing free cracks via bending metal coated film with a known curvature. To further enhance sensitivity and controllability, a guided crack formation is needed. Herein, we demonstrate such a ultra-sensitive sensor based on the guided formation of straight mechanical cracks. The sensor has patterned holes on the surface of the device, which concentrate the stress near patterned holes leading to generate uniform cracks connecting the holes throughout the surface. We found that such a guided straight crack formation resulted in an exponential dependence of the resistance against the strain, overriding known linear or power law dependences. Consequently, the sensors are highly sensitive to pressure (with a sensitivity of over 1 × 10^5^ at pressures of 8–9.5 kPa range) as well as strain (with a gauge factor of over 2 × 10^6^ at strains of 0–10% range). A new theoretical model for the guided crack system has been suggested to be in a good agreement with experiments. Durability and reproducibility have been also confirmed.

Studies on wearable healthcare and artificial electronic skin devices^1^,^2^,^3^,^4^ and the development of high performance sensors are attracting profound interests. Various types of pressure sensors have been developed based on nanowires^5^,^6^, silicon rubber^7^, piezoelectric^8^, and organic thin-film transistors^9^,^10^ for accumulating external information. Also, strain sensors based on carbon nanotubes^11^,^12^, nanofibres^13^, graphene platelets^14^ and mechanical cracks^15^,^16^,^17^ were reported. Although cracks were considered as defects to be avoided in general, studies on cracks such as patterning by cracks^18^, thin-film cracking for producing nanowires^19^, and interconnectors^20^,^21^ have been reported recently. A crack sensor inspired by the spider’s sensory system was reported to be remarkably sensitive to strain and vibration^15^. However, it has limitations in which strains of merely 2% can be applied. The cracks in Ref. 15 comprised a system of parallel cracks which revealed a universal behavior of the resistance against the strain which was close to a power-law dependence (see below). Yet, more control over the crack morphology and stretchability is still required for alternating the sensor into a more stretchable one for pressure sensing. Properly designed crack lip morphology can provide the rapid increase in the resistance with the strain and thus in sensitivity. Cracks should be made straight with negligible asperity that may give sharp lip disconnection and rapid increase in the resistance vs strain. In this work, we designed a micro-patterned crack sensor and demonstrated its ultrahigh sensitivity to external forces (e.g. pressure) and physiological signals (e.g., wrist pulse). An illustration of the patterned crack-sensor is presented in Fig. 1. We deposited stiff metal layers (first chromium 10 nm, then platinum 20 nm) on the top of a hole-patterned C-polyurethane acrylate^22^ (C-PUA) (details are in the Supplementary Experimental Section and Fig. S1). Then, 10% strain was applied to a rectangular shape sample along both grid directions one by one for crack formation. The lateral dimension of the sensor strip was 5 mm × 10 mm on the 100-μm-thick C-PUA as shown in Fig. 1a. In terms of the stretchability, the crack sensor can be stretched by 10% strain as shown in the photograph in Fig. 1b. Hole-patterns generate stress concentration between adjacent two holes and induce regular cracks as shown in Fig. 1a. When stretching force is applied to the patterned crack sensor, cracks formed perpendicular to the force are opened (red arrows) while the parallel ones are closed (blue arrows) because the Poisson’s ratio of rubber-like C-PUA induces such a behavior as shown in Fig. 1b. After preparing the crack network, the sensor undergoes the strain test. Before applying strain cracks are closed tightly as shown in Fig. 1c. After stretching the contact area between crack lips decrease as shown in Fig. 1d thus increasing the electrical resistance that was measured. The sketches in Fig. 1e and f explain a crucial difference between the free crack morphology and the guided straight crack morphology as to the number of contacts. From Fig. 1e it is seen that the contacts last longer while crack lips are being disconnected than for the straight crack case shown in Fig. 1f. This difference results in a different functional dependence of the resistance vs strain and different performance.

## Results

The resistance of the metal layer dramatically increased due to the opening of the cracks^15^ because there was no conductivity between disconnected lips of the crack. Rare, yet bridging, metal contacts between crack lips result in the high strain sensitivity of the resistance. The resistance analyzer measured the resistance of the metal layer on the polymer and the sensor was periodically stretched by the dynamic measurement system (3342 UTM, Instron Co.) as shown in Fig. 2a. Along with the high strain sensitivity, the crack-based sensor was demonstrated to be highly repeatable since the resistance variations of the sensor while loading up to 10% strain and unloading back to 0% strain at a sweeping speed of 10 mm/min (see Fig. 2b) closely match during the experiment. The overall gauge factor determined from the definition ((Δ*R/R*_0_)/*ε*) exceeds 2 × 10^6^ at strains of 0–10% as shown in Fig. 2c. It is noted that resistance variation to strain is non-linear (strain dependent gauge factor defined by [Gauge Factor = (*dR/R*_0_)/*dε*] is shown in Fig. S2). Cracks induced by hole-patterns showed regularity in shape, which results in reproducibility in five samples as shown in Fig. 2d. Graphs of the resistance of the patterned crack sensor loaded to strain 10% and unloaded to strain 0% are overlapped, which illustrates that some hysteresis occurs only at sufficiently high strains (see Fig. S3). In Fig. 2e we plot the experimental resistance vs strain curve along with theoretical fits that illustrates the validity of the theory given later. As to the response time, the cracks of the patterned crack sensor react within 100 ms to the abrupt sweep strain up to 0.4% (Fig. 2f). Finally, the marathon test has been performed for 5000 cycles (Fig. S4) to show good reproducibility even for strains up to 10%.

The initial resistance of the sensor without stretching is about 100 Ω. The maximum gauge factor is 2 × 10^6^ at strains of 0–10%. Consequently the resistance of the sensor varies from approximately 100 Ω to 20 MΩ. The power consumption of the sensor can be estimated with equation P = I^2^∙R, where I is the test current by the resistance analyzer (NI instruments, PXI-4071) and R is the resistance of the sensor. For the condition of the data acquisition system with highest resolution, the energy consumption of the sensor becomes 0.1 mW (test current is 1 mA, resistance of the sensor is 100 Ω, and resolution of data acquisition is 1 μΩ). With the lowest resolution, the power consumption would become 0.1 nW (test current is 1 μA, resistance of the sensor is 100 Ω, and resolution of data acquisition is 10 Ω). One can make an arrangement of the sensor strip by giving restriction to the ends of the crack sensor that can be used as a pressure sensor (Fig. 3e inset). Applying pressure makes the sample stretched and increases the resistance of the metal film. For the pressure measurements the crack sensor was mounted on a custom-built machine and resistance data were measured with the resistance analyzer (PXI-4071, National Instruments). Pressure data were acquired with a load cell (2712–041, Instron Co.). Resistance to pressure data obtained (Fig. 3a) could be approximately linearized into three pressure regions: (1) for 0–6 kPa the slope is 606.15 kPa^−1^, (2) for 6–8 kPa the slope is 40341.53 kPa^−1^, and (3) for 8–9.5 kPa the slope is 136018.16 kPa^−1^. These results exceed the performance of pressure sensitivities from previous studies at 6–9.5 kPa region. As shown in Fig. 3b, a tiny ant’s weight (Ponera japonica, 1 mg), corresponding to pressure 0.2 Pa, can be registered by the patterned crack sensor that illustrates the high performance of the pressure sensor. Physiological signals of wrist pulse can be measured by attaching the crack sensor to the wrist. As seen in Fig. 3d, detailed pulse data with percussion, tidal and diastolic waves can be easily resolved with high accuracy. To demonstrate the sensor scalability and ability to detect pressure with a spatial resolution, a sensing network of 16 pixels (4 × 4 pixel array) with dimensions of 6 × 6 cm^2^ is presented in Fig. 3e. The device, based on the crack sensor shows high flexibility and can be bent as given in Fig. 3f. Small LEGO pieces shaped as S, N, U was gently placed on the pixel arrayed sensor, while the induced pressure and their positions were easily detected as shown in Fig. 3h.

**Figure 1:**
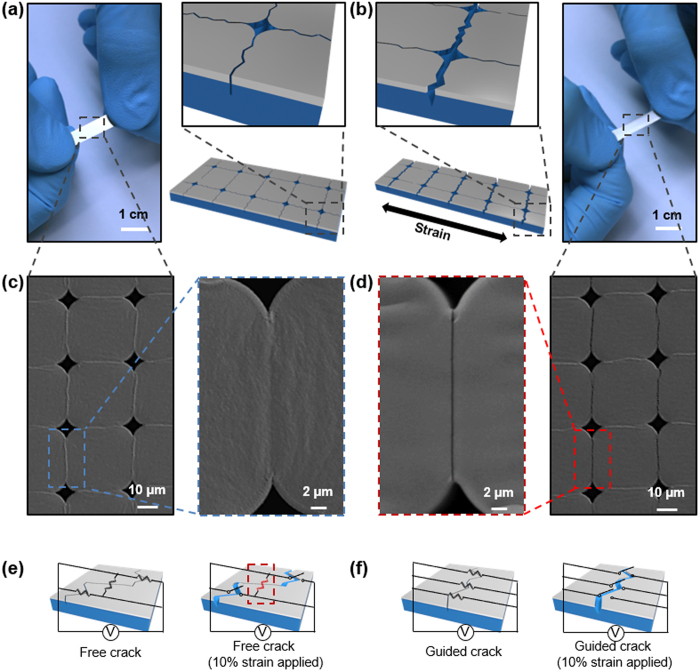
Schematic illustrations and images of an induced crack sensor. **(a)** Image (left) and illustrations (right) of the sensor before stretching. The sensor has lateral dimensions of 5 × 10 mm on 100-μm-thick C-PUA and all cracks are closed. **(b)** Image (right) and illustrations (left) of the sensor after stretching. Cracks perpendicular to the stretching direction are opened (red) and other ones are closed (blue). **(c)** SEM image of the sensor of the left image (left) of (a) and magnified SEM image of closed crack (right). **(d)** SEM image of the sensor of the right image (right) of (b) and magnified SEM image of opened crack (left). **(e,f)** Sketches of crack morphology free crack and guided crack case. Each cases are stretched to 10% strain (right). Resistances and switches represent electrical contacted and disconnected points. Red resistance inside the red dot square is a remained electrical contact point while the sensor is stretched to 10% strain.

**Figure 2:**
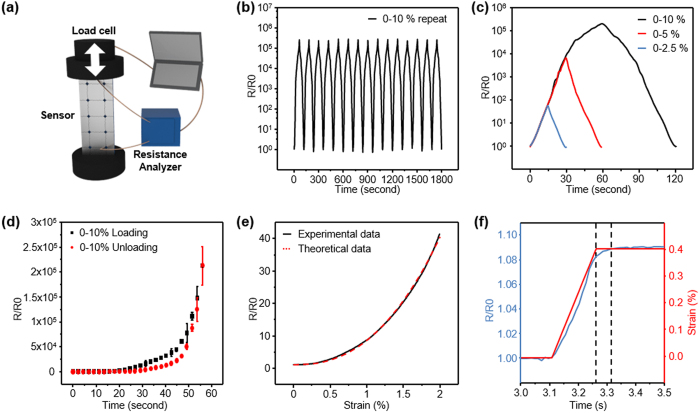
Resistance variations with strain of the crack sensor and theoretical analysis. **(a)** Illustration of the cyclic stretching test with a resistance analyzer. **(b)** The normalized resistance measured at a strain sweep rate of 10 mm/min. **(c)** loading/unloading behavior at various final strains. **(d)** loading/unloading behavior with five different samples for reproducibility. **(e)** Theoretical analysis data with experimental normalized resistance data. **(f)** Response time of the sensor resistance to strain 0.4%.

**Figure 3:**
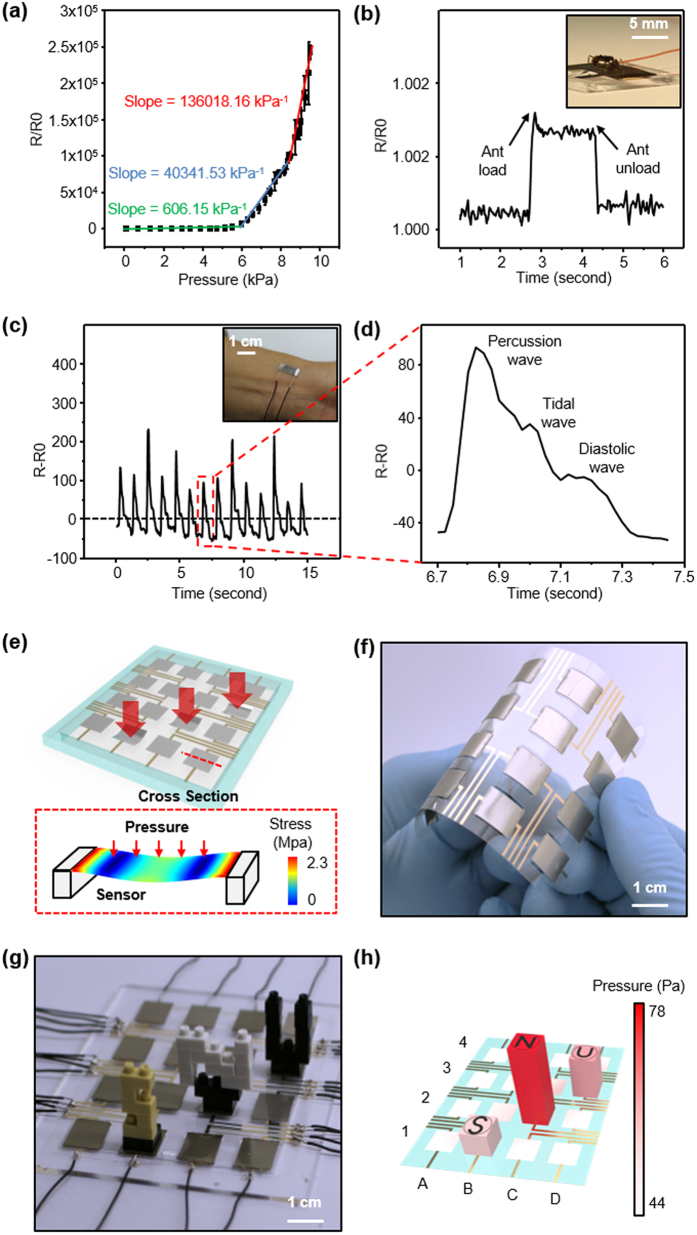
Resistance variations with pressure of the crack sensor, detecting physiological signal of wrist pulse and multi-pixel array of the crack sensor. (**a**) The normalized resistance data with pressure. Slope (green) is for 0–6 kPa, (blue) is for 6–8 kPa and (red) is for 8–9.5 kPa. (**b**) The induced crack sensor is able to sense very small pressures. Shown is the resistance change on placing and removing a small ant (1 mg) on the area of 50 mm^2^, corresponding to a pressure of 0.2 Pa. (**c**) Resistance change by a physiological signal of wrist pulse. Image of the sensor attached on wrist is shown in inset. (**d**) Typical characteristics of the wrist pulse including percussion, tidal and diastolic waves are shown. (**e**) The illustration of 8 × 8 array of the crack sensor, overall dimensions of the device are 6 × 6 cm^2^ and the pixel is 1 × 1 cm^2^. Inset is the image of FEM simulation showing stress distribution and deformation of the crack sensor with pressure. (**f**) Representative image of sensor flexibility. (**g**) Shown LEGOs are shaped as S, N, U placed on multi-array sensor. (**h**) Pressure distribution with pieces of LEGOs.

We performed a theoretical analysis of the resistance vs strain data that matches well the experimental data (Fig. 2e) for not too large stains. In our recent work^15^ we revealed a universal mechanism for a strain sensor based on parallel cracks in a cracked granular uniform 20 nm Pt film on a less stretchable polymer. Quite surprisingly, after we changed the uniform Pt film strip into a patterned one on a much more stretchable polymer (Fig. 1) of the present work, the strain dependence of the resistance dramatically switched from the *power-law* of Eq. (S6) into the *exponential* one in a much broader strain range up to 5% and more (See more details for theoretical analysis in Supplementary Information). One can notice the straight-line behavior up to 5% strain from the semi-log plot given in Fig. 2c.

**Figure 4:**
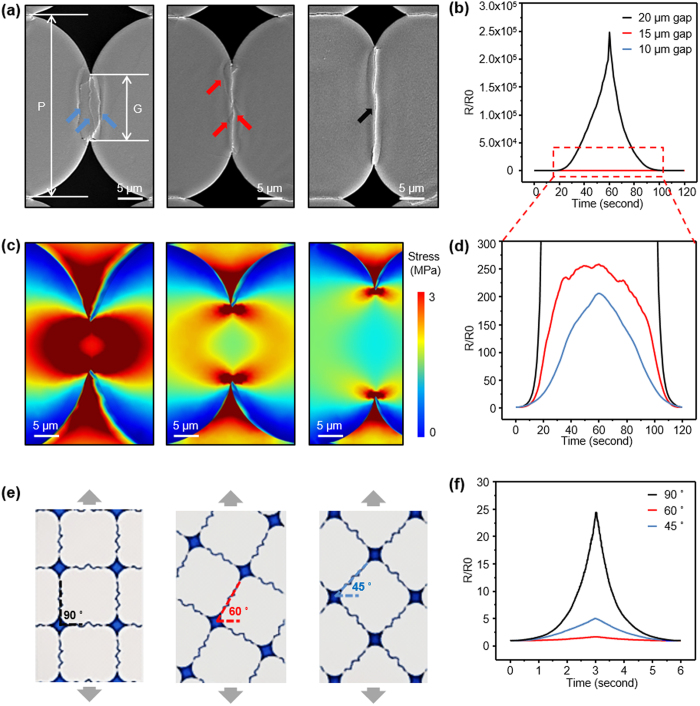
Resistance variation of induced crack sensor with three different hole-pattern and stretching angle. **(a)** SEM images of three different hole-pattern and shape of cracks. Arrows indicates each cracks. **(b,d)** 20 μm gap (black), 15 μm gap (red) and 10 μm gap (blue) sample normalized resistance data with samples stretched with strain 10%. **(c)** FEM modeling results of stress concentration on metal/polymer surfaces for different three hole-patterns. **(e)** Illustration of different pattern rotating angles. **(f)** Rotating angle of 90° (black), 60° (red), 45° (blue) sample normalized resistance data with samples stretched with strain 1%.

To confirm the validity of the theoretical assumptions, we investigate the effects of the crack morphology on resistance change while applying strain 10% to the crack sensor. First, we tested a sensor without any pattern. After performing the same crack inducing procedure, we obtained the crack network given in the SEM image in Fig. S5a. One can notice that the free cracks tend to have long straight parts perpendicular to the strain direction. Due to these parts the resistance vs strain curve should have the exponential behavior, according to the theory described above. We made sure that the experimental data for the non-patterned sensor given in Fig. S5b indeed follow the exponential law in a wide range of strain. However, in Fig. S5a one can notice a large amount of curved crack elements that deteriorate the sensor performance: The slope of the resistance vs strain curve is much lower than for the patterned crack sensor. In order to see the effect of the straight regions on the performance of the sensor three different hole-patterns were used. These patterns were characterized by pitches (P; the shortest distance between the hole centers) which were the same for all the three tested patterns and by the gaps (G; the shortest distance between the tips of holes) which were different (Fig. 4a). While stretching the crack sensor for crack generation, metal film was also stretched and fractured at the same time. The waves along the pattern gap were formed after the stretching force was removed. Different morphologies of waves were generated depending on the pattern gap, which were elliptic for the pattern gap of 10 μm, straightened for the pattern gap of 20 μm, and intermediate between elliptic and straight for the pattern gap of 15 μm. Only for the case of the pattern gap of 20 μm, a single straightened crack was formed between the pattern gap because the cracks were generated along the wave crests. Figure 4b shows the resistance change of the metal film while applying strain to the crack sensor. For non-straightened cracks, the gauge factor was demonstrated to be about 200, relatively low compared to that of the 20 μm pattern gap sample (over 2 × 10^6^). Non-straightened cracks have irregularity in their shape and behavior when strain is applied to the crack sensor. It happens that the elliptical shaped cracks could be also generated in the space 20 μm case. However, the portion of elliptical shaped cracks is small compared to straight-shaped cracks, therefore, its effect on sensor performance is negligible (Fig. S6). To understand this irregularity, we performed finite element method (FEM) simulations. Figure 4c illustrates the stress concentration at pattern gaps using FEM. The simulation result shows that narrower pattern gaps generate a wider distribution of high stress that stimulates appearing cracks everywhere within the gap distance. This is not the case for the 20 μm gap where the crack originates at the very tips of the gap (Fig. 4c, right plate). Additional confirmation of irregular morphology and opening of non-straightened cracks for narrower gaps, according to FEM simulations is given in Fig. S7. Thus the performance lower than for the patterned straight-crack sensor found above for the non-patterned crack sensor finds its explanation in the existence of multitude of curved cracks in the latter.

To additionally prove the viability of our theoretical concept of a single-parameter-dependent (which is the normalized gap size *x/x*_0_ = *k* ε/*x*_0_) resistance, we placed the square pattern at 60 and 45 degrees to compare the normalized resistance versus strain with the 90 degree case considered above (see the schematic in Fig. 4e). The experimental data are shown in Fig. 4f. Being re-plotted in log-log scale, the 60 degree curve closely coincides with the 90 degree curve after rescaling the strain by 0.32 (Fig. S8b). This is explained in the schematic in Fig. S8a by the effective narrowing of the relevant gap size due to a 90−60 = 30 degree geometry which brings the gap size at a given strain from *x* = *k* *ε* to sin(*π*/6)*x* = *k*(0.5*ε*) or even further to *k*(0.32*ε*) because of the additional contraction of the sample orthogonally to strain. Note that the gap of the complementary angle of 60 degrees has little relevance here because the conductivity is governed by the most conductive path which occurs through the narrower gap at 30 degrees. For 45 degree case with the same complementary angle the rescaling factor is 0.7, thus it is close to sin(*π*/4) = 1/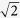 as it should be.

## Discussion

In conclusion, we report an ultrasensitive sensor based on a patterned crack metal film. The crack sensor exhibits ultrahigh gauge factors and can be applied as a multifunctional sensor for detecting strain, pressure and physiological signals. We revealed the underlying physical mechanism of exponential sensitivity and developed a theory which is consistent with the experiments. The reproducibility, flexibility and large-area coverage through multiplexing permit the integration of the device onto curvilinear human skin with advanced tools.

## Experimental Section

### Induced crack-based sensor fabrication

Spin coated 100 μm polydimethylsiloxane is bonded on glass by oxygen plasma (CUTE-1MPR, Femto Science Inc.). 20 μl polyurethane acrylate (PUA) dripped in PDMS/glass mold. Pillar patterned silicon mold cover on and 350 nm ultraviolet (UV) flood exposure (approximately 12 mJ/cm^2^) followed. A patterned 10 nm chrome layer is formed by thermal evaporator (thermal evaporator; Selcos Inc.) and 20 nm platinum layer with sputtering is followed. Metal layer deposited PUA film is carefully detached from PDMS/glass mold and stretched with 5% strain at x/y-direction by custom-built stretcher. Resistance change was measured by a Lab View-based PXI-4071 system (National Instruments Inc.).

### Multi-pixel array sample fabrication

To demonstrate the device’s scalability and ability for detecting mechanical vibrations and pressure, a sensing network of 16 pixels (4 × 4 pixel array) with an area of 6 × 6 cm^2^ is presented in Fig. 4f. The image and schematic view of the multi-pixel system are shown in Fig. 4e and g. Each pixel (1 × 1 cm^2^ islands) composed of 100-μm-thick PUA/10-nm-thick Cr/20-nm-thick Pt with hole-pattern, followed by stretching 10% bi-axially to generate cracks. The electrical connection between the cracked Pt and a Lab View-based PXI-4071 system (NI instrument Inc.) is formed by evaporated gold (Au, 50 nm thick) lines on PET film using shadow mask. Fabricated each pixel placed on PET film free standing and electrically connected to gold lines by conducting polymer (CW2400, circuitworks).

### Strain sensitivity measurement of crack sensors

The crack sensor is clamped by all-electric test instrument (3342 UTM, Instron Co.) and stretched by exact strain and force. Resistance change is measured by a Lab View-based PXI-4071 system (NI instrument). Various percentages of strain (2.5% to 10%) are applied to the sensor with speed (10 mm/min). Multiple-cycle tests (<10,000 cycles) with repeated loading-unloading of strain in the range of 0–2.5%, 0–5%, 0–10% and also use all-electric test instrument (3342 UTM, Instron Co.) with the speed of 10 mm/min.

### Pressure measurement of the crack sensor

The crack sensor is clamped by custom-built pressure test instrument, continuous pressure is applied to free standing crack sensor measured with load cell (2712-041, Instron Co.) and Lab View-based PXI-4071 resistance analyzer (NI instrument).

## Additional Information

**How to cite this article:** Choi, Y. W. *et al*. Ultra-sensitive Pressure sensor based on guided straight mechanical cracks. *Sci. Rep.* **7**, 40116; doi: 10.1038/srep40116 (2017).

**Publisher's note:** Springer Nature remains neutral with regard to jurisdictional claims in published maps and institutional affiliations.

## Supplementary Material

Supplementary Information

## References

[b1] Kim D.-H. . Epidermal electronics. science 333, 838–843 (2011). 21836009 10.1126/science.1206157

[b2] Viventi J. . Flexible, foldable, actively multiplexed, high-density electrode array for mapping brain activity *in vivo*. Nat Neurosci 14, 1599–1605, doi: 10.1038/nn.2973 (2011). 22081157 PMC3235709

[b3] Webb R. C. . Ultrathin conformal devices for precise and continuous thermal characterization of human skin. Nat Mater 12, 938–944, doi: 10.1038/nmat3755 (2013). 24037122 PMC3825211

[b4] Kim J. . Stretchable silicon nanoribbon electronics for skin prosthesis. Nat Commun 5, 5747, doi: 10.1038/ncomms6747 (2014). 25490072

[b5] Takei K. . Nanowire active-matrix circuitry for low-voltage macroscale artificial skin. Nat Mater 9, 821–826, doi: 10.1038/nmat2835 (2010). 20835235

[b6] Gong S. . A wearable and highly sensitive pressure sensor with ultrathin gold nanowires. Nat Commun 5, 3132, doi: 10.1038/ncomms4132 (2014). 24495897

[b7] Mannsfeld S. C. . Highly sensitive flexible pressure sensors with microstructured rubber dielectric layers. Nat Mater 9, 859–864, doi: 10.1038/nmat2834 (2010). 20835231

[b8] Pan C. . High-resolution electroluminescent imaging of pressure distribution using a piezoelectric nanowire LED array. Nature Photonics 7, 752–758, doi: 10.1038/nphoton.2013.191 (2013).

[b9] Zang Y. . Flexible suspended gate organic thin-film transistors for ultra-sensitive pressure detection. Nat Commun 6, 6269, doi: 10.1038/ncomms7269 (2015). 25872157 PMC4366495

[b10] Someya T. . A large-area, flexible pressure sensor matrix with organic field-effect transistors for artificial skin applications. Proc Natl Acad Sci U S A 101, 9966–9970, doi: 10.1073/pnas.0401918101 (2004). 15226508 PMC454198

[b11] Yamada T. . A stretchable carbon nanotube strain sensor for human-motion detection. Nat Nanotechnol 6, 296–301, doi: 10.1038/nnano.2011.36 (2011). 21441912

[b12] Park M., Kim H. & Youngblood J. P. Strain-dependent electrical resistance of multi-walled carbon nanotube/polymer composite films. Nanotechnology 19, 055705, doi: 10.1088/0957-4484/19/05/055705 (2008). 21817619

[b13] Pang C. . A flexible and highly sensitive strain-gauge sensor using reversible interlocking of nanofibres. Nat Mater 11, 795–801, doi: 10.1038/nmat3380 (2012). 22842511

[b14] Li X. . Stretchable and highly sensitive graphene-on-polymer strain sensors. Sci Rep 2, 870, doi: 10.1038/srep00870 (2012). 23162694 PMC3499758

[b15] Kang D. . Ultrasensitive mechanical crack-based sensor inspired by the spider sensory system. Nature 516, 222–226, doi: 10.1038/nature14002 (2014). 25503234

[b16] Wang Y. . Wearable and Highly Sensitive Graphene Strain Sensors for Human Motion Monitoring. Advanced Functional Materials 24, 4666–4670, doi: 10.1002/adfm.201400379 (2014).

[b17] Wang Y. . Ultra-sensitive graphene strain sensor for sound signal acquisition and recognition. Nano Research 8, 1627–1636, doi: 10.1007/s12274-014-0652-3 (2015).

[b18] Nam K. H., Park I. H. & Ko S. H. Patterning by controlled cracking. Nature 485, 221–224, doi: 10.1038/nature11002 (2012). 22575963

[b19] Adelung R. . Strain-controlled growth of nanowires within thin-film cracks. Nat Mater 3, 375–379, doi: 10.1038/nmat1128 (2004). 15133505

[b20] Graz I. M., Cotton D. P. J. & Lacour S. p. P. Extended cyclic uniaxial loading of stretchable gold thin-films on elastomeric substrates. Applied Physics Letters 94, 071902, doi: 10.1063/1.3076103 (2009).

[b21] Jones J., Lacour S. p. P., Wagner S. & Suo Z. Stretchable wavy metal interconnects. Journal of Vacuum Science & Technology A: Vacuum, Surfaces, and Films 22, 1723, doi: 10.1116/1.1756879 (2004).

[b22] Park J. . Conformal phase masks made of polyurethane acrylate with optimized elastic modulus for 3D nanopatterning. Journal of Materials Chemistry C 2, 2316, doi: 10.1039/c3tc32194k (2014).

