# Supplementary material for: Ultra-sensitive Pressure sensor based on guided straight mechanical cracks
Source: Sci Rep. 2017 Jan 6;7:40116. doi: 10.1038/srep40116 (PMC5216382; doi:10.1038/srep40116)
Supplement: Supplementary Information [file srep40116-s1.pdf]

Supplementary information

Ultra-sensitive Pressure sensor based on guided straight mechanical cracks

Yong Whan Choi[1] , Daeshik Kang[1,2], Peter V. Pikhitsa[1], Taemin Lee[1,3],
Sang Moon Kim[1,4] , Gunhee Lee[1,3],Dongha Tahk[3] , Mansoo Choi[1,3*]

[1]*Global Frontier Center for Multiscale Energy Systems,*
Department of Mechanical and Aerospace Engineering, Seoul National University, Seoul 151-742, Korea
[2]*Department of Mechanical Engineering, Ajou University, San 5, Woncheon-dong, Yeongtong-gu, Suwon 443-749, Republic of Korea*
[3]*Division of WCU Multiscale Mechanical Design, Department of Mechanical and Aerospace Engineering, Seoul National University, Seoul 151-742, Korea*
[4]*Department of Mechanical Engineering, Incheon National University, Incheon, 406-772,Korea*

**To whom correspondence should be addressed. E-mail:* mchoi@snu.ac.kr

Theoretical section

Theoretical Modeling

For that sensor due to a technology of producing large unidirectional strain the free cracks cut the sensor strip through so that the normalized conductance S of the sensor vs strain ε

$$S = \int_{\varepsilon}^{\infty} \mathbf{P}(x) dx \qquad \text{(S1)}$$

was determined by the probability distribution function (pdf) $\mathbf{P}(x)$ of the steps on a crack lip[15] making contacts between the lips. For a free crack we found an equation for $\mathbf{P}(x)$ with the only "size" parameter – the strain ε_0 that corresponds to the crack gap width $k\,\varepsilon_0$ being about the grain size $x_0 = k\,\varepsilon_0$

$$\mathbf{P}(x) = \mathbf{P}(1/x)/x^2 \ , \qquad \text{(S2)}$$

where $x = \frac{\varepsilon}{\varepsilon_0}$ and k is the proportionality factor to be defined by relating the crack gap width to the strain[15]. k can be different for different material realization of parallel crack systems and should be obtained from experiment.

Physically, Eq. (S2) states that tiny steps of the crack asperity made by the shifts of grains are distributed the same as the large steps made by grain piling, because the substrate elastic field being scale-less and thus having no characteristic length may not distinguish between tiny and large meandering asperity. Among solutions of Eq. (S2) one may choose either the *log-normal* pdf

$$\mathbf{P}(\varepsilon) = \frac{1}{\varepsilon\mu\sqrt{\pi}} \exp\left(-\frac{(\ln(\varepsilon/\varepsilon_0))^2}{\mu^2}\right) \qquad \text{(S3)}$$

or a nearly identical *log-logistic* pdf

$$\mathbf{P}(\varepsilon) = \frac{B}{\varepsilon_0} \frac{(\varepsilon/\varepsilon_0)^{B-1}}{(1+(\varepsilon/\varepsilon_0)^B)^2} \qquad \text{(S4),}$$

where μ and B are parameters of the pdf.

Both of the distributions of Eqs. (S3) and (S4) belong to the class of so-called skew distributions with long tails. As we discussed in Ref. 1, the non-zero probability of large but rare contacts between crack lips lies in the essence of the mechanism of the conduction through the crack and is therefore in concordance with the tailed distributions. With Eq. (S3), Eq. (S1) gives for the resistance $R = 1/S$ as a function of strain the following:

$$R = 2/\left(1 - \mathrm{erf}\left(\frac{\ln\left(\frac{\varepsilon}{\varepsilon_0}\right)}{\mu}\right)\right) \quad , \tag{S5}$$

$\mathrm{erf}(x)$ is the error function. Eq. (S5) renders the normalized resistance that remarkably fits the experiment[15] for the strains up to 2%. At the same time, one can show that the log-logistic pdf of Eq. (S4) together with Eq. (S1) leads to

$$R = 1 + (\varepsilon/\varepsilon_0)^B \tag{S6}$$

that fits the experiment[15] with fitting parameters $\varepsilon_0 = 0.39$ and $B = 2.39$ (see Fig S12) with the same accuracy as the log-normal pdf of Eq. (S5). Yet, the *power-law* function of Eq. (S6) is much simpler than the error function in Eq. (S5). We may suggest this universal power law for data fitting by experimentalists who study free parallel cracks.

Quite surprisingly, after we changed the uniform Pt film strip into a patterned one on a much more stretchable polymer (Fig. 1) of the present work, the strain dependence of the resistance dramatically switched from the *power-law* of Eq.(S6) into the *exponential* one in a much broader strain range up to 5% and more. One can notice the straight-line behavior up to 5 % strain from the semi-log plot given in Fig 2c. Here we discuss the underlying mechanism of this phenomenon.

A crucial difference of the cracks generated in the current study from those in the previous study[15] is shown in Fig S10 b,c: the cracks between pattern patches closely follow the "crests" of the wrinkles on the metal/polymer film. That means that the crack

path was extremely directed and only close neighboring Pt grains were disconnected along the crack lip (Fig S11). In this respect, the local deviations are about the size of a grain and thus, may not satisfy the scaling Eq. (S2) for free crack generation. On the other hand, the pattern patches were pressed to each other in the horizontal and perpendicular direction to the strain direction as shown in Fig S10b, because of the Poisson ratio of 0.5[2] which is an inherent characteristic of rubber-like materials. Therefore, the system remained unchanged effectively in one dimension with cut-through cracks that are now located on a train of squares lined up in the horizontal direction (Fig S10).

In analogy with the study[15], it is sufficient to calculate the step pdf. According to Fig S11, along the lip of a crack (the crack trajectory) each ith grain can stay shifted up or down (in the strain direction) with probability ½ and shift y_i. The crack step size means the shifting distance of the trajectory upwards (downwards) by several neighboring grains. As seen in Fig S11, the sum of, say, three grain shifts, made in one direction, produces the step of size x. Assume that the local grain shifts are distributed with a local pdf $P(y)$. Neighboring grains of normalized size 1 vertically shifted in small steps $y_1, \dots, y_n$ (Fig S11) should have the global pdf $\mathbf{P}(x)$ of the step size x

$$\mathbf{P}(x) = \iiint_0^1 \sum_n \delta(y_1 + \cdots + y_n - x) \frac{1}{2^n} P(y_1) \dots P(y_n) d y_1 \dots dy_n \tag{S7}$$

where

$$\int_0^1 P(y) d y = 1 \tag{S8}$$

δ is the delta-function, and $n = 1,2, \dots$ The delta-function presents the microscopic pdf of a step to be constructed of n positive shifts in one direction satisfying the equation $y_1 + \cdots + y_n - x = 0$. As we assumed, the probability of shifting a grain up (down) is ½. Therefore, defining the step as the total shift up, the probability of a given

configuration with small steps of n should be proportional to $\frac{1}{2^n}$. The details of further integration of Eq. (S7) in the complex plane are given in the Supplementary Information (Theoretical section). Conveniently, by closing the integration contour of the corresponding Cauchy integral in Eq. (S4) by an infinitely large semicircle in the lower half plane (Fig S12), one gets $\mathbf{P}(x)$ as a sum of the residues at poles, and the largest of the exponential terms, governed by the pole $-iz_0$, where z_0 is a positive real number, will be dominating at large x. Then if one restricts oneself by this pole, one gets the normalized probability

$$\mathbf{P}(x) = \exp(-z_0 x)\, z_0 \quad . \tag{S9}$$

It is clear from Eq. (S1) that the conductance S will also be the exponential function of strain at large strains,

$$S = \int_{\varepsilon}^{\infty} \exp(-z_0 x)\, z_0 dx = \exp(-\mathrm{z}_0 \varepsilon) \tag{S10}$$

as well as the resistance

$$R = 1/S \; = \; \exp(\mathrm{z}_0 \varepsilon) \equiv \exp(\varepsilon/\varepsilon_0). \tag{S11}$$

One can see the difference between Eq. (S6) and Eq. (S11), the *power-law* and the *exponential*. A quite general example of $P(y) = 1$ that assumes arbitrary grain positions with respect to each other neighbor and then, a homogeneous distribution of the grain shifts along the crack lip in Fig S11, gives the dominating pole of $z_0 = 1.256$ (see the Supplementary Information and Fig S12**)**. In Fig 2e we give the normalized resistance vs strain calculated with $P(y) = 1$ (the red line) along with the pure exponential function of Eq. (S11) (the green line in Fig 2e) to see a close coincidence between the experimental data and the theory.

While fitting the experiment with, for example, the resistance vs strain calculated with the uniform pdf of grains with the asymptotic function Eq. (S11), one had to rescale the strain by $\alpha = 0.7$ times to match the linear slope of the experiment in Fig 2c. Physically that means that we restrict the shift of the grains by 30% and thus flatten the crack lips. The resistance therefore responses to such a flattening by increasing the slope of the resistance in the semi-logarithmic scale. Parameter α measures the degree of flatness of the crack lip. From Fig S11 one can notice that the maximum slope of the step asperity is restricted by α which is the tangent of the maximum slope angle. In case of $P(y) = 1$ the maximum slope angle is 45 degree with the tangent $\alpha = 1$. Of course if the crack lips were completely flat with no shift and $\alpha = 0$, then we would have a sudden disconnection of the lips and the infinite slope of R/R_0. According to our fitting in Fig 2e, the parameter $\varepsilon_0 = \frac{\alpha}{z_0} = \frac{0.7}{z_0} \approx 0.6$ for the strain measured in %. With these estimations at hand we can calculate the characteristic grain size x_0 . As far as SEM images reveal the gap distance x to be proportional to the strain $x = k\,\varepsilon$, where $k \approx 50$ nm with ε in %, then the grain size $x_0 = k\,\varepsilon_0 \approx 30$ nm, quite close to the constituent primary particle size of the granular Pt film.

Complex integration

Then, after rewriting δ - function as a Fourier integral, Eq. (7) reads,

$$\mathbf{P}(x) = 1/2\pi \int_{-\infty}^{\infty} \iiint_0^1 \sum_n \exp(i\alpha(y_1 + \cdots + y_n - x)) \frac{1}{2^n} P(y_1) \ldots P(y_n) d\,y_1 \ldots dy_n \; d\alpha$$

(S12)

or after simplification of Eq. (S12) due to the independent integration over each of y_i

$$\mathbf{P}(x) = 1/2\pi \int_{-\infty}^{\infty} \sum_n \exp(-i\alpha x) \left[\frac{1}{2} f(\alpha)\right]^n \; d\alpha \qquad \text{(S13)}$$

where

$$f(\alpha) = \int_0^1 P(y)\exp(i\alpha y)dy \ . \qquad \text{(S14)}$$

The geometrical series of Eq. (S2) can be directly transformed into

$$\mathbf{P}(x) = 1/2\pi \int_{-\infty}^{\infty} \exp(-i\alpha x)\frac{\frac{1}{2}f(\alpha)}{1-\frac{1}{2}f(\alpha)}d\alpha \qquad \text{(S15)}$$

The Cauchy integral in Eq. (S15) can be analyzed in general terms. One can notice that the decay of function $\mathbf{P}(x)$ at large x may be nearly exponential and nearly independent of a particular form of $P(y)$:

$$\mathbf{P}(x)\sim \exp(-z_0 x) \ \text{ for } x \gg 1 \ \text{ and } \ z_0 > 0 \qquad \text{(S16)}$$

if there is the dominating role of one pole in the denominator of Eq. (S4)

$$1 - \frac{1}{2}f(-iz_0) = 0 \qquad \text{(S17)}$$

with the lowest *real* $z_0 > 0$ in Eq. (S6). All other poles (solutions of Eq. (S17) should be complex and also lie in the lower half of the complex plane (see the example in Fig S12). One can see from Eq. (S14) that such a single pure imaginary pole $\alpha = -iz_0$ should always exist because otherwise it is impossible to make the integral in Eq. (S14) equal to 2 while having the pole in the upper half. Indeed, if $\alpha = iz_0$, then $|\exp(i\alpha y)| = |\exp(-z_0 y)| \leq 1$ and it is impossible to make the integral $f(\alpha)$ of Eq. (S14) larger than 1, because the integrand contains the normalized probability function and even if $|\exp(i\alpha y)|$ were exactly 1 for all y Eq. (S3) would give at maximum only 1. But then Eq. (S17) cannot be satisfied, because it demands that $f(\alpha) = 2 > 1$.

Consider a quite general example of $P(y) = 1$ that assumes arbitrary grain positions with respect to each other neighbor and then, a homogeneous distribution of the grain shifts along the crack lip in Fig S12. For this case Eq. (S14) gives:

$$f(\alpha) = (\exp(i\alpha) - 1)/i\alpha \qquad \text{(S18)}$$

and then Eq.(S17) takes the form:

$$2z_0 + 1 - \exp(z_0) = 0. \tag{S19}$$

Solutions of Eq. (S18) can be found numerically. The lowest $z_0 = 1.256$ and the other poles are $2.789 \pm 7.438i,\ 3.360 \pm 13.866i$ .... (see Fig S12).

Estimating the low end detection limit

It is observed that the maximum resistance change by strain 0.04 % was 2.1468 Ω, and the noise level was below 0.7037 Ω. Signal-to-noise ratio become as ~3, so the low end detection limit is 0.04/3 = 0.013%.

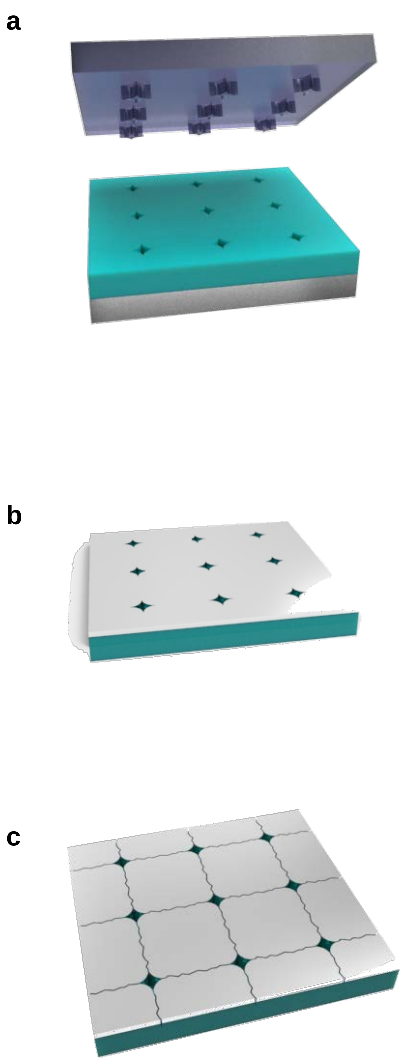

Figure S1. Fabrication procedure for induced crack sensor. (a) Dripping PUA on glass and cover with silicon mold. UV exposure is followed for curing PUA. **(b)** Metal layer (Cr 10 nm/Pt 20 nm) is deposited. **(c)** 10 % of strain is applied bi-axially.

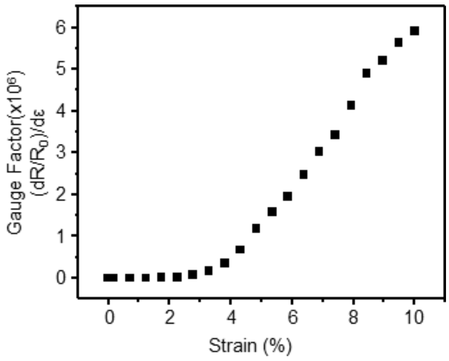

Figure S2. Strain-dependent gauge factor by taking the derivative of R/R_0 with respect to strain [Gauge Factor = $(dR/R_0)/d\varepsilon$].

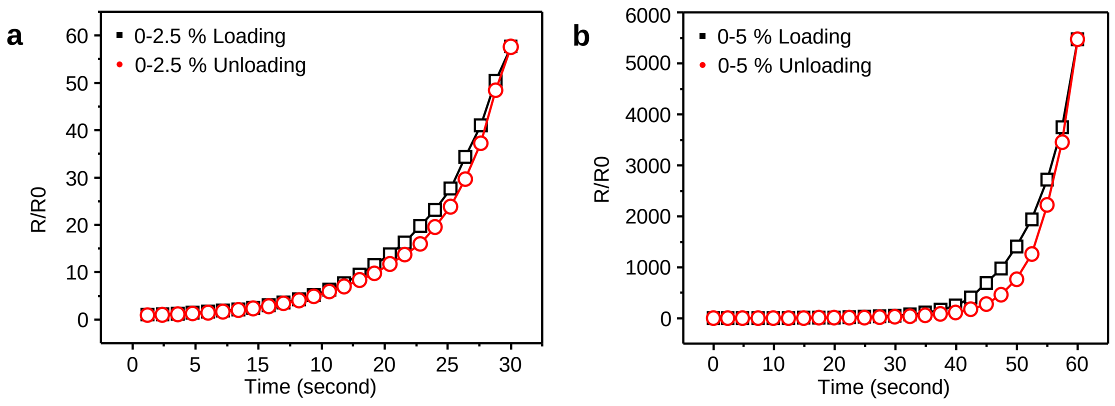

Figure S3. Loading/unloading data of various strain applied. (a) Reversible loading/unloading behaviour at final strain 0.25 %. (b) Hysteresis of loading/unloading at final strain 5 %.

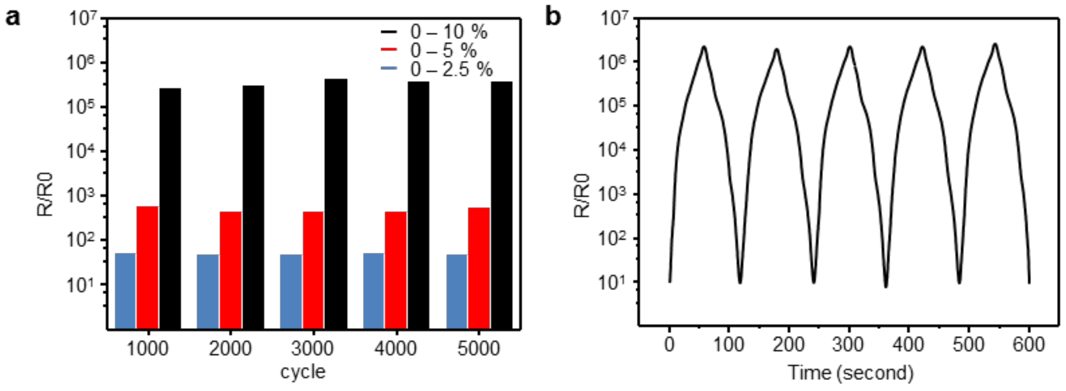

Figure S4. Marathon tests (<5,000 cycles) with repeated loading/unloading of various final strain. (a) Marathon test with various final strain (black – at final strain 10 %, red – at final strain 5 %, blue – at final strain 2.5 %) (b) Loading/unloading at 10 % final strain after 5,000 cyclic test.

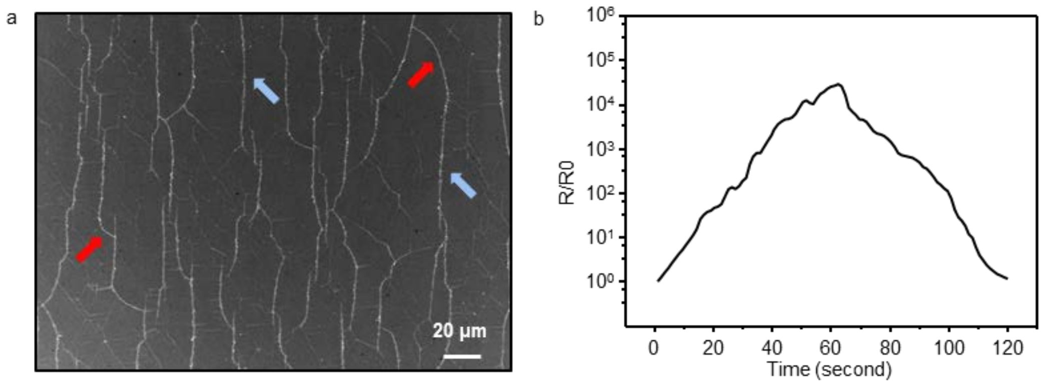

Figure S5. Non-patterned sensor. (**a**) SEM image of cracks. (Red arrows: curved crack, Blue arrows: straight crack) (**b**) The normalized resistance vs strain of non - patterned sensor.

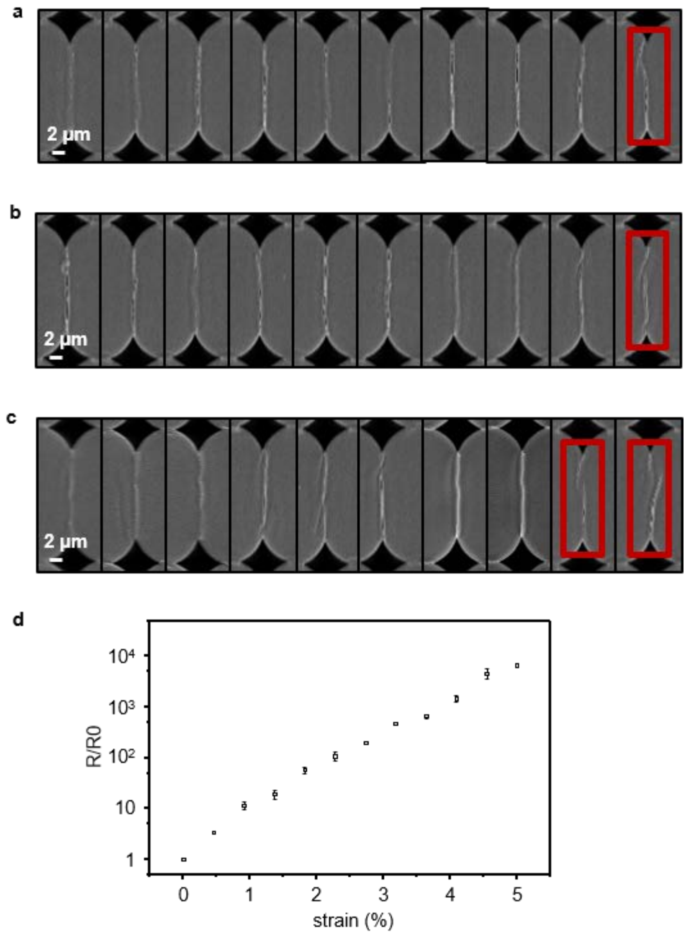

Figure S6. Variation of formed cracks on three different sets of samples (a,b,c). Ten SEM images for each sample shows that only one or two elliptical-shaped cracks from ten cracks are formed. (red square indicates the elliptical shaped cracks). **(d)** The normalized resistance variation with respect to strain measured upto 5 % final strain for three different samples.

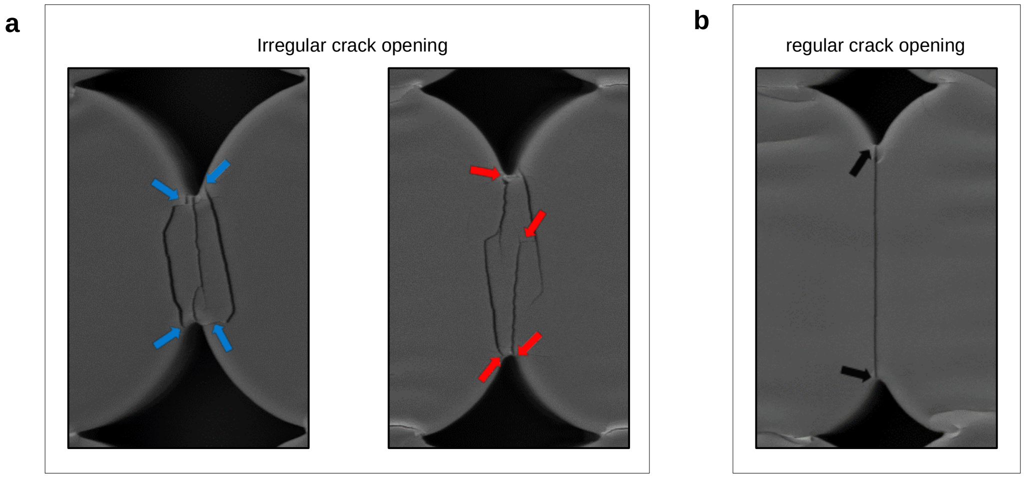

Figure S7. Crack morphologies vary to different hole pattern. (a) Irregular crack formations for pattern gap 10 µm and 15 µm. Blue-arrow indicates contact points left of pattern gap 10 µm with 10 % strain applied. Red-arrow is for pattern gap 15 µm. **(b)** Straight crack formation for pattern gap 20 µm. Black-arrow indicates contact points left.

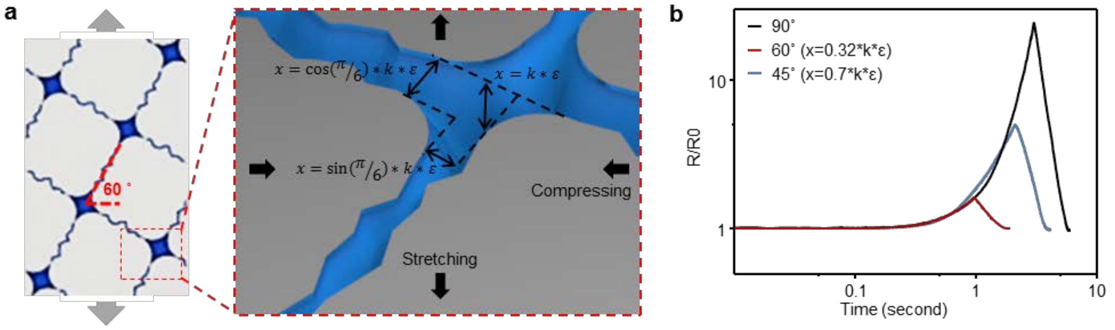

Figure S8. Relevant crack opening by rotating pattern at 60 degrees. (a) Schematic of crack opening by rotating pattern at 60 degrees. **(b)** log-log scale re-plotting of Fig 3f showing close coincidence of the graphs after the corresponding abscissa rescaling.

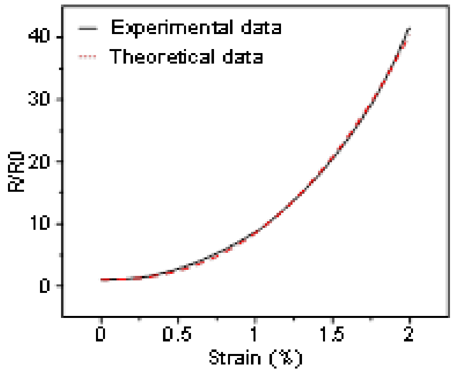

Figure S9. The normalized resistance vs strain. The power dependence of Eq. (6) (red line) compared with the data from our previous work [2] with a parallel crack sensor.

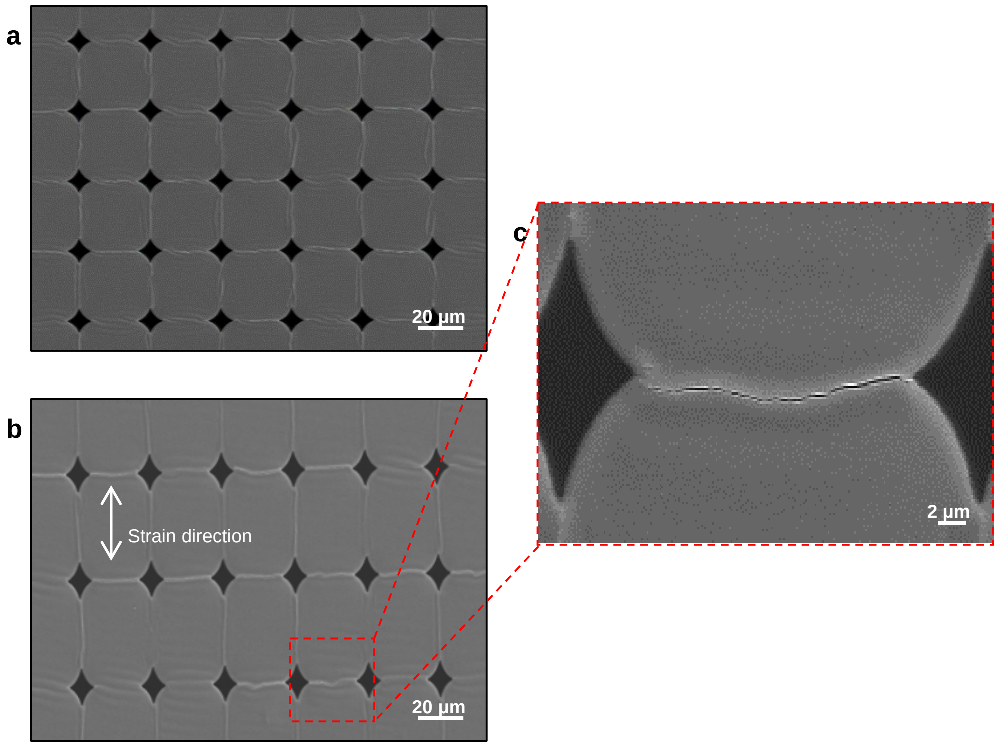

Figure S10. SEM image of crack formation on metal film. (a) SEM image of crack within 0 % strain. **(b)** SEM image of crack within 10 % strain. **(c)** Crack formation on crest between hole gap..

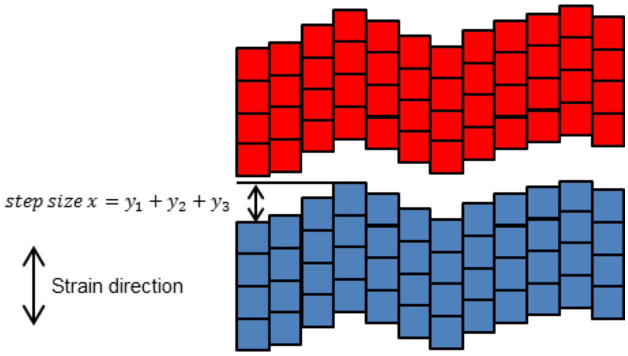

Figure S11. Lips of a crack modelled with the grains of size 1. The step size determines the connection-disconnection events between the opposite lips.

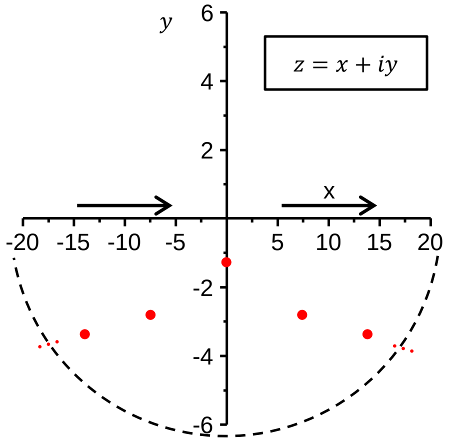

Figure S12. Complex plane part encircled by the integration contour.

No.	Active materials	minimum detection	maximum detection	sensitivity	ref
**1**	PSR nanowire (NW)-FET	0.5 kPa	15 kPa	11.5 μS kPa^{-1}	**[6]**
**2**	Gold NWs	13 Pa	50 kPa	1.14 kPa^{-1}	**[7]**
**3**	PDMS microstructure OFET	3 Pa	20 kPa	0.55 kPa^{-1}	**[8]**
**4**	Suspended gate OFET	0.5 Pa	20 kPa	158.6 kPa^{-1}	**[9]**
**5**	Pressure-sensitive rubber (PSR) OFET	10 kPa	30 kPa		**[10]**
**6**	this work	**0.2 Pa**	10 kPa	**136018 kPa^{-1}**	

Table S1. A chart of transparent strain sensors' specifications of strain range and gauge factor.

No.	Active materials	maximum detection	Gauge Factor	ref
**1**	Cracked metal film	2%	2000	[1]
**2**	ZnO NWs array	0.8%	1813	[3]
**3**	Single ZnO fine wire	1.2%	1250	[4]
**4**	Single $ZnSnO_3$ NW	0.32%	3740	[5]
**5**	This work	10%	**50000** (at 5% strain)	

Table S2. A chart of transparent pressure sensors' specifications of pressure range and sensitivity.

References

1 Kang, D. *et al.* Ultrasensitive mechanical crack-based sensor inspired by the spider sensory system. *Nature* **516**, 222-226, doi:10.1038/nature14002 (2014).

2 Brown, R. *Physical testing of rubber*. (Springer Science & Business Media, 2006).

3 Zhang, W., Zhu, R., Nguyen, V. & Yang, R. Highly sensitive and flexible strain sensors based on vertical zinc oxide nanowire arrays. *Sensors and Actuators A: Physical* **205**, 164-169 (2014).

4 Wu, J. M. et al. Ultrahigh sensitive piezotronic strain sensors based on a ZnSnO3 nanowire/microwire. *ACS nano* **6**, 4369-4374 (2012).

5 Zhou, J. et al. Flexible piezotronic strain sensor. *Nano letters* **8**, 3035-3040 (2008).

6 Takei, K. et al. Nanowire active-matrix circuitry for low-voltage macroscale artificial skin. *Nature materials* **9**, 821-826, (2010).

7 Gong, S. et al. A wearable and highly sensitive pressure sensor with ultrathin gold nanowires. *Nature communications* **5**, 3132, (2014).

8 Mannsfeld, S. C. et al. Highly sensitive flexible pressure sensors with microstructured rubber dielectric layers. *Nature materials* **9**, 859-864, (2010).

9 Zang, Y. et al. Flexible suspended gate organic thin-film transistors for ultra-sensitive pressure detection. *Nature communications* **6**, 6269, (2015).

10 Someya, T. et al. A large-area, flexible pressure sensor matrix with organic field-effect transistors for artificial skin applications. *Proceedings of the National Academy of Sciences of the United States of America* **101**, 9966-9970, (2004).
